# Considering Burnout and Well-Being: Emergency Medicine Resident Shift Scheduling Platform and Satisfaction Insights from a Quality Improvement Project

**DOI:** 10.3390/healthcare12060612

**Published:** 2024-03-08

**Authors:** Jamaji C. Nwanaji-Enwerem, Tori F. Ehrhardt, Brittney Gordon, Hannah Meyer, Annemarie Cardell, Maurice Selby, Bradley A. Wallace, Matthew Gittinger, Jeffrey N. Siegelman

**Affiliations:** 1 Department of Emergency Medicine, Emory University School of Medicine, Atlanta, GA 30322, USA; 2 Gangarosa Department of Environmental Health, Emory Rollins School of Public Health, Atlanta, GA 30322, USA

**Keywords:** AI, wellness, ACGME, medical education, burnout, MedEd

## Abstract

Few studies explore emergency medicine (EM) residency shift scheduling software as a mechanism to reduce administrative demands and broader resident burnout. A local needs assessment demonstrated a learning curve for chief resident schedulers and several areas for improvement. In an institutional quality improvement project, we utilized an external online cross-sectional convenience sampling pilot survey of United States EM residency programs to collect information on manual versus software-based resident shift scheduling practices and associated scheduler and scheduler-perceived resident satisfaction. Our external survey response rate was 19/253 (8%), with all United States regions (i.e., northeast, southeast, midwest, west, and southwest) represented. Two programs (11%) reported manual scheduling without any software. ShiftAdmin was the most popularly reported scheduling software (53%). Although not statistically significant, manual scheduling had the lowest satisfaction score and programs with ≤30 residents reported the highest levels of satisfaction. Our data suggest that improvements in existing software-based technologies are needed. Artificial intelligence technologies may prove useful for reducing administrative scheduling demands and optimizing resident scheduling satisfaction.

## 1. Introduction

A recent survey employing the validated and widely used Maslach Burnout Inventory demonstrated that over 75% of emergency medicine resident physicians experience burnout—an occupational phenomenon defined by the World Health Organization as a syndrome “resulting from chronic workplace stress that has not been successfully managed” [1,2,3,4]. The etiology of burnout is multifactorial, but it is inherently both an occupational patient safety and physician wellness issue. Thus, efforts to address burnout should be a public health priority. One notable contributor to burnout among emergency medicine residents is shift scheduling, with 98% of residents in one survey identifying shift schedules as a key factor in their overall wellness [5]. Although the United States Accreditation Council for Graduate Medical Education (ACGME) sets duty hour guidelines for emergency medicine residents, beyond these guidelines, residency programs have freedom to schedule their residents to meet their institutional needs [6]. Still, these institutional needs must be balanced with requested schedule preferences and often result in weeks or more of manual effort by schedulers who are often overtaxed chief residents.

**Figure 1:**
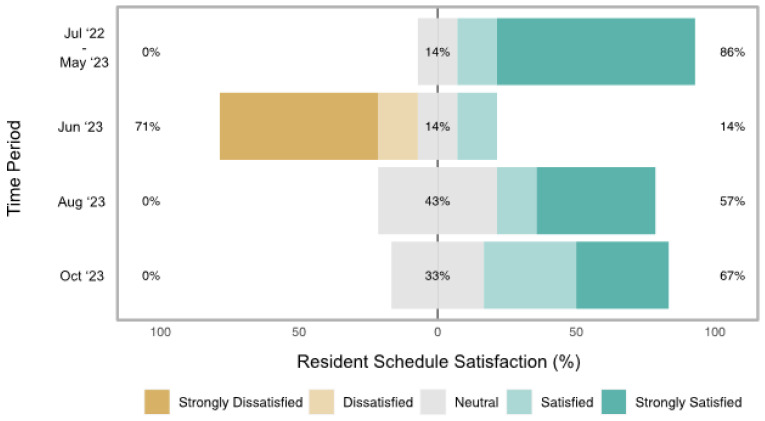
Resident reported schedule satisfaction from the needs assessment survey. This figure presents a centered horizontal bar plot of internal program resident schedule satisfaction throughout the quality improvement study period. June 2023 satisfaction (the first schedule made by new chief residents) was significantly lower (*p* = 0.01) than academic year July 2022–May 2023 satisfaction (schedules made by old chief residents). Subsequent schedules created by the new chief residents achieved greater resident satisfaction, as August 2023 (*p* = 0.02) and October 2023 (*p* = 0.01) resident satisfaction was statistically greater than baseline June 2023 resident satisfaction. There was no statistically significant difference in resident satisfaction in August 2023 (*p* = 0.28) and October 2023 (*p* = 0.23) compared to academic year July 2022–May 2023, suggesting that new chiefs achieved satisfaction levels comparable to the old chiefs. *p*-Values from Mann–Whitney U tests.

Such was the experience in our residency program, and our study was created as part of an initiative aimed at improving the quality and equity of our resident scheduling practices. Our program is a three-year ACGME-accredited emergency medicine training program with 19 residents per year. Annually, four senior residents are chosen to be chief residents, with resident schedule creating among their key job duties, and they are on-boarded by the outgoing chief residents over a period of months. Like previous years, our chief residents of the 2023–2024 academic year faced a learning curve when they began their term, anecdotally spending many hours on schedule creation, with mixed effects on resident satisfaction. Our chiefs use the MedRez platform, which includes features like shift templating, an automation feature, and the ability to block off requested days and highlight duty hour violations. Nevertheless, we found that the automation function did not perform well for our program, resulting in our chiefs finding it more convenient to manually schedule shifts with the MedRez templates. Our program also has set rules that residents follow when submitting schedule requests; still, there remained individual scheduler idiosyncrasies. An internal survey was conducted as a needs assessment and highlighted both the scheduler learning curve and several specific areas for improvement, as shown in Figure 1 and Table 1.

We wondered if other residency programs around the nation were dealing with similar challenges and if there was a way to further standardize the scheduling process to benefit residents and the schedulers. Previous studies have explored resident preferences with respect to factors including shift length, circadian scheduling, and sequential versus split night shifts, but to the best of our knowledge, there are no data examining the modalities used to build emergency medicine resident schedules and satisfaction related to those respective scheduling modalities [5,7,8]. To address this gap, we analyzed data gathered as part of a quality improvement pilot survey to understand what scheduling modalities/software are used by United States emergency medicine residencies and the levels of scheduler satisfaction with the scheduling modalities. Understanding that satisfaction is multifactorial, including variables like ease of schedule creation for the scheduler and alignment with individual preferences of the schedule consumers, we hypothesized that—when they work—automated scheduling practices may better account for these variables and mitigate administrative burdens, thereby yielding higher resident and scheduler satisfaction when compared to manual scheduling.

## 2. Methods

### 2.1. Needs Assessment

As a needs assessment, we conducted an anonymous Google Form baseline satisfaction survey of our upper-level residents (*n* = 32) posted in our residency SMS group chat in May of 2023. This survey had four components. First, we asked residents to rate their satisfaction with schedules made by outgoing chief residents during the 2022–2023 academic residency year on a Likert scale from 1 (strongly unsatisfied) to 5 (strongly satisfied). Second, residents were also given a free response section to list pain points with the 2022–2023 schedules. Third, in the same baseline survey, residents were asked to rate their satisfaction with the June 2023 schedule created by the incoming chief residents and, fourth, asked to provide free response pain points with the June 2023 schedule. Excluding the December schedule, for which we have different holiday scheduling rules, follow-up surveys were sent to residents at two month increments to track satisfaction for the August and October 2023 schedules. Residents were sent reminders to complete the surveys via the same SMS group chat, and the response rate for the baseline survey served as the target response rate for subsequent surveys. Our baseline needs assessment survey had a response rate of 7/32 (22%). Response rates for the August and October surveys were 7/32 (22%) and 9/32 (28%), respectively.

### 2.2. Study Design

We performed a convenience sampling pilot survey of United States ACGME-accredited emergency medicine residency programs (*n* ≈ 253). Data about satisfaction with resident scheduling platforms and formats were collected via an anonymous 9-item survey designed by our emergency medicine program leadership. The survey was pilot tested with program directors at other sites, with feedback used to edit questions for clarity. The survey was uploaded to Qualtrics (Qualtrics International Inc., Seattle, WA, USA) and distributed to program directors through the Council of Residency Directors in Emergency Medicine (CORD) email listserv in the summer of 2023 with instructions to have program leadership or chief residents complete it. The survey included a combination of free-response and multiple-choice items including the size of the residency program (≤30 residents or >30 residents), if programs conducted their resident scheduling manually or with a specific software, and particular pain points with their current scheduling practices (Table 2). Overall scheduler and scheduler-perceived resident satisfaction with current scheduling practices were collected on a Likert scale from 1 (strongly unsatisfied) to 5 (strongly satisfied). Given the exploratory nature of this external survey, we did not have an a priori survey response goal. This project met the criteria for non-human subject research based on the Emory University Institutional Review Board determination form.

### 2.3. Data Analysis

We examined relationships of satisfaction with scheduling software/platforms/characteristics using Mann–Whitney U tests. Manual scheduling, scheduling practice length <2 years, and programs with ≤30 residents served as the references. Relationships between scheduler and scheduler-perceived resident satisfaction were evaluated using Spearman correlations. Survey questions without answers were coded as “Unknown.” All statistical analyses were performed using R Version 4.2.2 (R Core Team, Vienna, Austria) and a *p*-value < 0.05 was used as the threshold for statistical significance.

## 3. Results

Our external survey response rate was estimated at 19/253 (8%). Twelve surveys (63%) were completed by program directors, four (21%) were completed by assistant/associate program directors, one (5%) was completed by a chief resident, one (5%) was completed by a program coordinator, and one (5%) person did not specify their program role. The responses represented all United States regions (i.e., northeast, southeast, midwest, west, and southwest). Ten programs (53%) were responsible for regularly scheduling more than thirty residents. Only two programs (11%) reported scheduling manually without any special software. Of the manually scheduled programs, one did not report their program size, while the other reported scheduling for less than 30 residents. ShiftAdmin was the most popular scheduling software used (53% of respondents).

Scheduler and scheduler-perceived resident satisfaction were modestly correlated (Spearman coefficient = 0.38). Table 3 presents the results of the platform and characteristic relationships with satisfaction. Scheduler satisfaction and scheduler-perceived resident satisfaction had overall means (medians) of 3.4 (3) and 3.2 (3), respectively. Manual scheduling had the lowest scheduler-perceived resident satisfaction score when compared to utilizing scheduling software and individual software platforms. Programs scheduling for 30 or less residents reported the highest levels of scheduler satisfaction and scheduler-perceived resident satisfaction. In the platform analysis, scheduler satisfaction was greatest with MetricAid, ShiftAdmin, and Qgenda/ShiftAdmin. In the format analysis, excluding the program that did not report their scheduling format, scheduler satisfaction was greatest with software scheduling when compared to manual scheduling. None of these relationships reached the threshold for statistical significance. Four programs reported using their scheduling platform for less than two years and were using manual scheduling, MetricAid, Qgenda, and ShiftAdmin, respectively. Five programs reported using their scheduling platform for greater than six years. Of these programs, one was scheduling manually and the remaining four used ShiftAdmin. Both resident and scheduler satisfaction were lowest in programs using their platforms for less than two years. Again, these relationships did not reach the threshold for statistical significance.

Free-response questions demonstrated software cost, long-term program use, and the same platform used for faculty/attending scheduling as the primary reasons for which the programs decided to use a specific software platform. Common pain points included suboptimal automation algorithms given the complexity of scheduling even after meeting with software representatives, steep learning curves that new chief residents must learn each year, and cost. Respondents also pointed to residency program features such as chronic understaffing, scheduling residents at multiple sites, scheduling residents of different training levels, and unlimited resident day-off requests as factors that further complicated the scheduling process.

## 4. Discussion

In this quality improvement project and convenience sample pilot survey, we examined the relationships of scheduling platforms/characteristics with scheduler and scheduler-perceived resident satisfaction. The results from our internal needs assessment demonstrated that residents became more satisfied with chief resident schedules over time. Our external survey demonstrated that manual scheduling was consistently ranked the poorest in terms of scheduler-perceived resident satisfaction and was among the bottom-ranked platforms in terms of scheduler satisfaction. We also observed higher scheduler-perceived resident and scheduler satisfaction in programs incorporating scheduling software, programs with thirty or less residents, and programs using their scheduling platform for more than two years. 

We prioritized completing this project in our chief year because of the literature that consistently ranks emergency medicine amongst the medical specialties with the highest levels of physician burnout [9,10]. Thus, finding evidence-based and tangible means of optimizing residents’ schedule preferences and mitigating the burden of administrative tasks like scheduling has immense potential to improve overall physician wellness [11]. We utilize the MedRez platform, which offers an automation scheduling feature. However, when trialed with rules to optimize the feature for the specific demands of our program, sub-optimum schedules were created and required burdensome manual edits. Instead, we have found it easier to utilize some of the software’s features (i.e., blocking out day-off requests, creating tallies for keeping track of certain kinds of shifts, and placing alerts for duty hour violations), but perform most of the scheduling manually. In our experience, it takes a chief resident 40–60 h in total per month to complete an upper-level schedule. Our needs assessment demonstrated that with time, residents became more satisfied with their clinical schedules. This is likely due to our chiefs becoming more familiar with the nuances of making schedules, but also likely attributable to discussions facilitated through our monthly chief-led meetings during resident conference didactics. These meetings served as an opportunity to review ACGME guidelines and already existing institutional schedule request rules (e.g., on emergency department months, residents can request four days off but only three can be contiguous and at most three can be Saturdays or Sundays) that were published on our residency shared drive. Residents were pleased at efforts made to give them at least one calendar day off following an overnight shift. Still, many expressed that they would prefer to only have the 24 h period off if they had to choose between sacrificing one of their requested days off. The meetings also allowed for discussions about scheduling practices that could benefit from change but would be difficult to change. For example, our chiefs have no control over our pediatric emergency department shift schedule. As such, finding ways to limit two strings of night shifts in these months remains a work in progress. 

Although our external program survey results do not meet statistical significance, likely due to our small sample size, we believe that they provide insights for improving resident scheduling practices and an impetus for further research in this area. Although scheduler and scheduler-perceived resident satisfaction appear to be average overall, other programs—including those incorporating scheduling software—described issues with learning curves and meeting complex program-specific needs even after meeting with software representatives for scheduling optimization. This echoes our program’s experience and suggests that baseline scheduling software offerings can be improved and may be an underappreciated target for emerging artificial intelligence technology applications in emergency medicine [12]. Such technology may help to mitigate the administrative burden on chief resident schedulers while also mitigating the drop in resident schedule satisfaction that happens yearly in our program and possibly in other programs across the nation.

While we do have residency program representation from each United States region, our study is limited by its small sample size. Our external survey aimed to address ways of improving the continued administrative burden of schedule-making, but was limited by a poor survey response rate (8%). Only two programs reported strictly manual scheduling, and there were no statistically significant findings related to satisfaction and scheduling platforms/characteristics. Still, we believe that the results remain informative as a pilot study and the only emergency medicine study of its kind that we could identify through a literature review. Furthermore, we recognize the need for more comprehensive research in this area and hope that our pilot study can help inform these efforts. Finally, our external survey relied on the perceptions of residency leaders responding to the survey and no resident non-schedulers were surveyed. Directly surveying resident non-schedulers in all survey instruments will be critical in future work. In addition to utilizing larger sample sizes, future studies may also benefit from more comprehensive analyses of scheduling software platform features and an evaluation of long-term impacts of scheduling practices. 

## 5. Conclusions

In summary, we report findings of scheduler and scheduler-perceived resident satisfaction with emergency medicine resident scheduling platforms and characteristics. Although not statistically significant, these latter data suggest that existing scheduling software needs to be improved and that there may be resident and scheduler benefits to using scheduling software. Ultimately, our work can help to inform more comprehensive studies as well as efforts aimed at tackling physician wellness by optimizing scheduling practices. 

## Figures and Tables

**Table 1:** Pain-point comments from the internal resident satisfaction needs assessment survey free responses.

Easy to get burnt out if you have several evening shifts in a row.
Difficult to transition to overnight shifts with no calendar day off in between the day shift and night shift.
Wellness can be compromised when you have to go directly from an off service rotation into the next rotation (I’m sure this is out of your control though).
When prior chiefs made schedules, they considered shifts surrounding requests off. This schedule does not do that (i.e., trying to leave for a weekend and I got it off but I work 3–11 on Friday and work backup Monday making travel very difficult).
Would prefer multiple days in a row and having extended 3 days off in a row.
Would prefer longer stretches of shifts with longer stretches of days off rather than multiple strings of just 2–3 shifts with one day off in between, but that may just be personal preference.
Wish I had more grouped breaks and not just one day off at a time.
It’s a little brutal to have 2 sets of night shifts on split adult and pediatric months. I’m not sure if there’s a work around or a way to create balance between our schedules.
Finished overnights at the end of September and then switched back to nights again 1st week of October.

**Table 2:** External national ACGME-accredited schedule satisfaction survey content.

What program/software does your residency program use for making resident schedules? (Enter n/a if you do not subscribe to a scheduling software program)
2. Within this software do you schedule? Manually Using Software Automation Other
3. How many years have you been using this method? <2 years 2–4 years 4–6 years >6 years
4. What made you choose this scheduling software? (free response)
5. Rate scheduler satisfaction with your current scheduling program/software. 1 (least) = Strongly Unsatisfied. 5 (most) = Strongly Satisfied Scheduler = the faculty member(s) or chief resident(s) who make the clinical schedule
6. To the best of your ability, rate resident satisfaction with their schedules produced using this method. 1 (least) = Strongly Unsatisfied. 5 (most) = Strongly Satisfied
7. Please describe any pain points with your current scheduling program/software. (free response)
8. What is your role in the program? (free response)
9. How many residents are in your program? ≤30 >30

**Table 3:** Relationships of schedule platforms and characteristics with scheduler and scheduler-perceived resident satisfaction from an external pilot survey.

	Scheduler Satisfaction Mean (Median)	*p*-Value	Scheduler-Perceived Resident Satisfaction Mean (Median)	*p*-Value
**Overall**				
Study Sample (*n* = 19)	3.4 (3)	-	3.2 (3)	-
**Scheduling Platform**				
Manual (*n* = 2)	3 (3)	reference	2.5 (2.5)	reference
MedRez (*n* = 2)	3 (3)	0.99	3.5 (3.5)	0.67
MetricAid (*n* = 1)	4 (4)	0.99	4 (4)	0.99
Qgenda (*n* = 3)	2.3 (3)	0.99	3 (3)	0.99
ShiftAdmin (*n* = 10)	3.7 (3.5)	0.82	3.3 (3)	0.73
Qgenda/ShiftAdmin (*n* = 1)	4 (4)	0.99	4 (4)	0.99
**Scheduling Format**				
Manual (*n* = 2)	3 (3)	reference	2.5 (2.5)	reference
Software (*n* = 16)	3.3 (3)	0.99	3.3 (3)	0.66
Unknown (*n* = 1)	5 (5)	0.99	5 (5)	0.67
**Scheduling Practice Length**				
<2 years (*n* = 4)	2.8 (3)	reference	2.8 (3)	reference
2–4 years (*n* = 7)	3.3 (3)	0.63	3.7 (4)	0.28
4–6 years (*n* = 3)	3.3 (3)	0.69	3.3 (4)	0.58
>6 years (*n* = 5)	4 (4)	0.20	3 (3)	0.79
**Number of Residents**				
≤30 (*n* = 4)	3.75 (3.5)	reference	4 (4)	reference
>30 (*n* = 10)	3.2 (3)	0.50	3.4 (3.5)	0.25
Unknown (*n* = 5)	3.4 (3)	-	2.4 (2)	-

*p*-values from Mann–Whitney U tests for manual scheduling, scheduling practice length <2 years, and programs with ≤30 residents serve as the references.

## Data Availability

Data are contained within the article.
